# Draft Genome Sequence of an Escherichia coli Sequence Type 420 Isolate from a Patient with Urinary Tract Infection in Northern California

**DOI:** 10.1128/MRA.00251-20

**Published:** 2020-06-04

**Authors:** Yusuke Matsui, Yuan Hu, Nicole J. Tarlton, Lee W. Riley

**Affiliations:** a School of Public Health, Division of Infectious Diseases and Vaccinology, University of California, Berkeley, Berkeley, California, USA; b School of Public Health, Division of Epidemiology, University of California, Berkeley, Berkeley, California, USA; University of Maryland School of Medicine

## Abstract

The genome sequence of a uropathogenic Escherichia coli sequence type 420 strain isolated from a patient with urinary tract infection in northern California is described here. The draft genome sequence includes a 4.8-Mb chromosome, accompanied by a 114-kb plasmid containing IncFIB/IncFII/Col156 and a 35-kb plasmid containing IncN3.

## ANNOUNCEMENT

Extraintestinal pathogenic Escherichia coli (ExPEC) belonging to multilocus sequence type 420 (ST420) is often reported as a low-prevalence genotype (1, 2), but it has been identified sporadically from patients with community-acquired urinary tract infection (UTI) at a college campus in northern California (3–5). Interestingly, all of those ST420 isolates were found to be susceptible to all antimicrobial agents tested (pan-susceptible) (4, 5). Here, we present the genome sequence of an ST420 ExPEC strain isolated from one of the patients (a 39-year-old female) diagnosed with UTI at the university health center in 2003 (4). In this paper and in GenBank, this ST420 isolate is designated strain IT0021. The genome sequence was determined for future use as a reference sequence.

We isolated E. coli from a urine sample demonstrating a viable bacterial count of >10^4^ CFU/ml, as described preciously (4, 6). *E. coli* was subcultured from a frozen glycerol stock and grown overnight in LB broth in a 37°C shaker. The next day, 2 ml of culture was pelleted in a microfuge tube, and the pellet was subjected to DNA extraction. DNA purification for whole-genome sequencing was performed with the DNeasy blood and tissue kit (Qiagen). Preparation of Illumina-compatible libraries for 300-bp paired-end reads was conducted according to a standard protocol (WaferGen Bio-systems). Libraries were sequenced on a MiSeq instrument with MiSeq reagent kit v3 (600-cycle) chemistry. Read trimming and contig assembly and analysis were performed with Geneious v.9.0.5 (Biomatters, Ltd.) (7). RAST was utilized for annotation of the draft genome (8). Plasmids, resistance genes, and putative virulence factor genes were initially identified with Web-based tools from the Center for Genomic Epidemiology (http://www.genomicepidemiology.org), and prophages were identified with PHASTER (9). Default parameters were used for all software unless otherwise specified.

We assembled 509,467 of 514,170 reads with the Geneious assembler. We set the threshold value of base call confidence as high quality. The E. coli IT0021 draft genome sequence was assembled into 133 contigs >200 bp long, with a mean contig length of 47,293 bp, a maximum contig length of 410,189 bp, and an *N*_50_ of 89,241 bp. The mean read coverage of the assembled contigs was approximately 25-fold. The contigs comprised a 4,695,334-bp chromosome, a 114,729-bp circular plasmid, and a 35,105-bp circular plasmid. The GC contents were 50%, 51%, and 51%, respectively.

The chromosome contained four prophages, namely, PhiV10, SfΙ, mEp460, and lambda. The 114-kb plasmid contained IncFIB/IncFII/Col156 replicons. It closely resembled pUTI89* (pSF-166-1, >99% assembled), which was found in an ST95 ExPEC isolate (10). Most ST95 strains are pansusceptible to antibiotics, and the carriage of pUTI89* is statistically significantly associated with antibiotic susceptibility (11). The plasmid carried *senB* (11), which encodes an enterotoxin, but no antibiotic resistance genes. The 35-kb plasmid contained an IncN3 replicon and closely resembled pTRE-131 (>95% of pTRE-131 assembled) (12). It contained no virulence genes or antibiotic resistance genes.

### Data availability.

This whole-genome shotgun project has been deposited in DDBJ/ENA/GenBank under the accession number JAAMRS000000000. The version described in this paper is version JAAMRS010000000. Raw sequence reads have been deposited in the NCBI Sequence Read Archive under BioProject number PRJNA607787 and run number SRR11277725.
